# Comparison of handcrafted features and convolutional neural networks for liver MR image adequacy assessment

**DOI:** 10.1038/s41598-020-77264-y

**Published:** 2020-11-23

**Authors:** Wenyi Lin, Kyle Hasenstab, Guilherme Moura Cunha, Armin Schwartzman

**Affiliations:** 1 Division of Biostatistics, Department of Family Medicine and Public Health, University of California San Diego, La Jolla, 92093 USA; 2 Department of Mathematics and Statistics, San Diego State University, San Diego, CA 92182 USA; 3 Liver Imaging Group, Department of Radiology, University of California San Diego, La Jolla, 92093 USA; 4 Halıcıoğlu Data Science Institute, University of California San Diego, La Jolla, 92093 USA

**Keywords:** Hepatology, Magnetic resonance imaging

## Abstract

We propose a random forest classifier for identifying adequacy of liver MR images using handcrafted (HC) features and deep convolutional neural networks (CNNs), and analyze the relative role of these two components in relation to the training sample size. The HC features, specifically developed for this application, include Gaussian mixture models, Euler characteristic curves and texture analysis. Using HC features outperforms the CNN for smaller sample sizes and with increased interpretability. On the other hand, with enough training data, the combined classifier outperforms the models trained with HC features or CNN features alone. These results illustrate the added value of HC features with respect to CNNs, especially when insufficient data is available, as is often found in clinical studies.

## Introduction

Deep learning methods are becoming increasingly popular because of their impressive classification performance. However, it is known that they typically require a large training sample to achieve that accuracy. Meanwhile, handcrafted (HC) features have been implemented for decades and still serve as a powerful tool when combined with machine learning classifiers. Could HC features be preferable, especially if the training sample is small? The answer to this question cannot be answered in great generality but depends on the context. In this paper, we investigate this question with respect to a specific medical image analysis problem, namely that of identifying adequacy of contrast-enhanced liver MR images.

**Figure 1:**
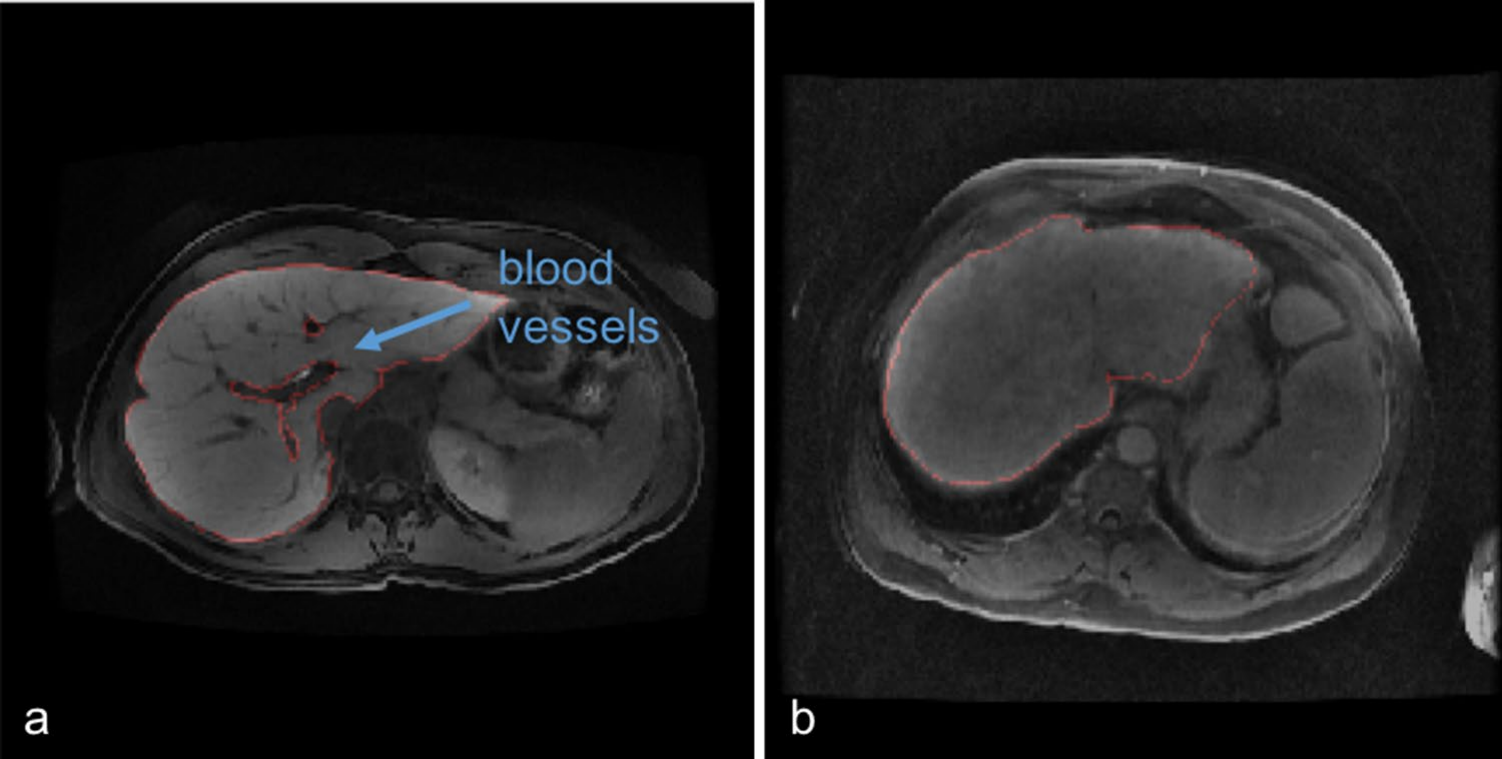
HBP MR images acquired at 20 min after the injection of hepatobiliary contrast agent and corresponding segmented liver regions (red line) (**a**) Adequate HBP: vessels are hypointense to background liver and clearly visible. (**b**) Suboptimal HBP: vessels are isointense to liver and difficult to identify.

Hepatobiliary phase (HBP) magnetic resonance imaging (MRI) with intracellular contrast is routinely performed to detect and characterize focal liver lesions^1^. On adequate HBP images, intravenous contrast uptaken by the liver cells causes blood vessels and most lesions to appear dark relative to the bright background liver, which facilitates lesion detection (Fig. 1a). Adequate HBP can occur between 10 and 60 min post-contrast depending on patient physiology and liver function. However, images acquired too early after injection may have insufficient contrast in the liver, resulting in impaired differentiation between normal liver tissue and focal lesions (i.e. suboptimal HBP)^2^. Similarly, liver dysfunction can impair contrast uptake and produce images where the background liver has similar intensity to blood vessels and lesions^3^, rendering images suboptimal for lesion detection and characterization (Fig. 1b). Since timing of the acquisition may affect the diagnostic value of HBP images, real-time assessment of HBP adequacy could improve diagnostic assessment and workflow efficiency by individually tailoring exams’ length to the liver’s ability to uptake contrast. Therefore, there is an interest in developing machine learning approaches for automatically classifying liver MR images as either having suboptimal or adequate HBP.

Supervised learning algorithms, i.e., learning a mapping from input data to output (labels) from a set of training examples, are widely used in medical image data analysis^4,5^. Traditional supervised learning algorithms, such as random forest (RF), support vector machines (SVM) and k-nearest neighbors, require prespecified HC features, while Deep Convolutional Neural Networks (CNNs) learn image features from the inputs to classify labels. However, CNNs typically require large numbers of training examples, which can be difficult to obtain in the medical imaging space^6^, due to confidentiality constraints, financial limitations and time required for expert annotations. Thus, traditional methods using HC features remain useful in dealing with limited samples of medical imaging data. For example, in the work of Nakanishi et al.^7^, a bootstrap aggregated random forest was used to assess image quality (IQ) of coronary computed tomography angiography. Pizarro et al.^8^ applied a support vector machine to automatically rate the quality of 3D brain MRI. These works exemplify the feasibility of feature-driven classifiers but do not consider CNNs as alternatives.

Considering the evaluation of HBP adequacy, radiologists routinely check liver image quality for various quality-related factors via visual inspection. However, as image acquisition is performed by MRI technicians who have limited expertise for assessing HBP adequacy at the time of the examination, suboptimal images may only be recognized many hours after the examination is completed by the radiologist in the reading room. This may result in impaired accuracy of the study or the need to recall the patient, which is costly and inconvenient. Esses et al. implemented a deep learning approach using a CNN for image quality evaluation of T2-weighted liver acquisitions, which is a fully automated procedure without any HC features^9^. However, this data-driven process only achieved an accuracy of 80% and the manner in which results were achieved had limited interpretability.

Research has shown deep neural networks require a relatively large number of training examples to achieve high accuracy, but changes in predictive performance and its relation to sample size is not thoroughly discussed. Luo et al. explored the effect of training sample size on CNN-based network performance and concluded that larger training sets improve classification performance^10^. To better explore the question of adequate training sample size, we compare the performance of HC features and CNN with varying sample sizes.

In this paper, we propose methods for classification using HC features specifically developed for assessing liver MR image adequacy, and analyze the role that these HC features play in relation to CNNs and training sample sizes. We show that using HC features outperforms the CNN across smaller sample sizes and with increased interpretability. We also show that with enough training data, the proposed classification model trained on both HC and CNN features outperforms the models trained with HC features or CNN features alone. These results suggest that, without enough data, such as at the early stage of a new study, machine learning algorithms using HC features may be a more viable choice. These could be complemented with CNNs once more data become available for the study.

## Methods

**Figure 2:**
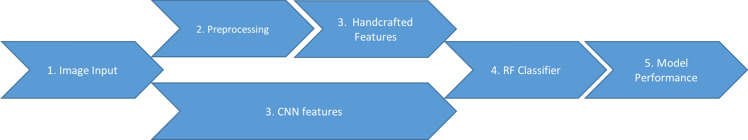
Study pipeline of evaluating adequacy of liver MR images.

In this work, we developed a supervised learning approach for determining adequacy of HBP liver images using the analysis pipeline outlined in Fig. 2. With acquired HBP liver MR images in step 1, each of the 3-dimensional (3D) liver MR image series was preprocessed in step 2 to extract HC features in step 3. Alternatively, CNN features were directly extracted from the original image inputs using a CNN model. In step 4, a RF classifier was used to classify the MR image series with derived features. Classification performance was evaluated using radiologist annotated ground-truths in step 5.

### Data source

The imaging data comprises 1201 T1-weighted 3D HBP MR image series from 406 patients who underwent Gd-EOB-DTPA-enhanced liver MRI. Two liver expert radiologists individually classified each image series as adequate or suboptimal HBP. Discordant classifications were adjudicated after further inspection in consensus. In the end, the 1201 liver MR images were classified into 902 adequate cases and 299 suboptimal cases. Among the 406 patients, 70% of the patients (826 images) were randomly assigned as the full-size training data and the remaining were assigned as testing data. This retrospective Health Insurance Portability and Accountability Act (HIPAA)-compliant study was approved by the institutional review board (IRB) of prospective and retrospective observational study in human subjects undergoing radiology examinations for clinical care (HRPP# 171538) with waived written informed consent. The data collection and all experiments were performed in accordance with the relevant guidelines and regulations.

### Preprocessing

In the remainder of the methods, $I_i (x, y, z)$ denotes the intensity at coordinates (*x*, *y*, *z*), in the acquired 3D liver MRI sample *i*. All 1201 images were preprocessed using the publicly available software Advanced Normalization Tools (ANTs; http://www.pic-sl.upenn.edu/ANTs) and its python package known as ANTsPy.

In order to focus the input data on the organ of interest, we segmented the liver using an independently developed 2D liver segmentation CNN with U-Net model architecture^11^. Slices of 3D HBP images were individually propagated through the segmentation network and concatenated to form 3D binary masks. By multiplying intensities of the original liver MR images with their corresponding binary masks, only signal intensities inside the liver mask area were saved for the following analysis (intensity liver masks). Only the 10 middle slices of each liver MRI were stored to increase computational speed during subsequent preprocessing and feature extraction.

**Figure 3:**
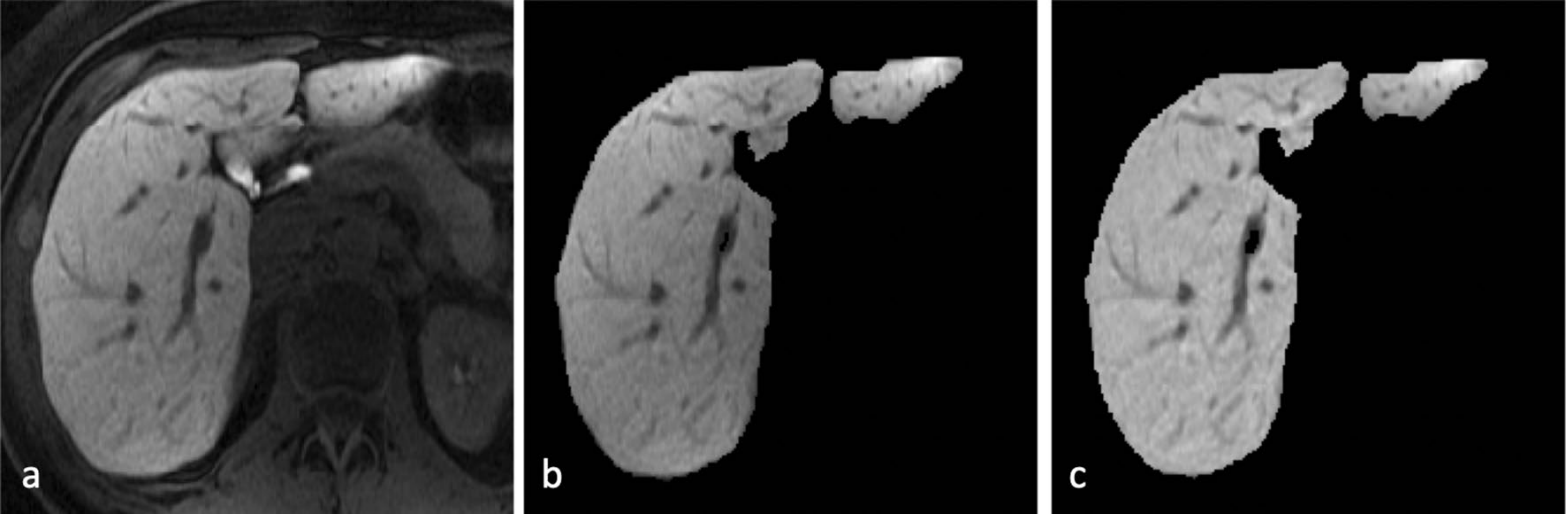
An example of liver MRI preprocessing (**a**) raw image (**b**) liver segmentation (**c**) noise reduction and background inhomogeneity correction.

A nonparametric nonuniform normalization (N3) approach, called N4ITK^12^, was performed to remove intensity inhomogeneity artifacts. Compared with the original N3 method, N4ITK uses an advantageous B-spline smoothing strategy, which has better performance. The image was then convolved with a Gaussian kernel to reduce image noise and the segmented liver boundary was binarily eroded to exclude artifacts attributed to the Gaussian smooth. In order to have a standardized imaging space, intensity values were normalized to have mean 0 and standard deviation 1 across all voxels inside the liver mask by subtracting the mean intensity values and dividing by their standard deviations. An example of the preprocessing procedure is shown in Fig. 3.

### Feature extraction

Three categories of HC features were taken into consideration: intensity values, topological structure and texture information. HC features were extracted from the preprocessed images and subsequently used as inputs to a RF classifier. HC features were also used with automatically generated CNN features in the RF classifier.

#### Gaussian mixture model (GMM)

Intensity separation is achieved using Gaussian Mixture Models (GMMs). Generally speaking, a mixture model is a probabilistic modeling tool for separating subgroups within an overall population. Although different types of distributions can be used in the mixture, the Gaussian models are most commonly applied in image intensity separation because of their simplicity^13^.

The main objective is to separate a grayscale liver MR image consisting of *N* voxels into 2 classes ($\Delta =1,2$). The 2-component GMM assumes that the probability density function of a voxel intensity $I_i$ is $P(I_i=x_i) = (1-\pi )g_1(x_i)+\pi g_2(x_i)$, where $\pi$ is the probability of the voxel intensity $I_i$ belonging to the second class and $g_1$, $g_2$ are Gaussian densities with parameters $(\mu _1,\sigma _1)$ and $(\mu _2, \sigma _2)$ respectively. The log-likelihood based on *N* voxels is given by

$$\begin{aligned} \sum _{i=1}^{N}\log \{(1-\pi )g_1(x_i)+\pi g_2(x_i)\} \end{aligned}$$.

The expectation–maximization (EM) algorithm is an iterative algorithm used to estimate the parameters of the component densities by the method of maximum likelihood^14^. Each iteration *t* consists two steps:

- E-step: for each voxel $I_i$, compute the posterior probability,
  $$\begin{aligned} p^{(t)}(\Delta =2|I_i)=\frac{\pi ^{(t)}g_2(I_i|\mu _2^{(t)},\sigma _2^{(t)})}{(1-\pi ^{(t)})g_1(I_i|\mu _1^{(t)},\sigma _1^{(t)})+\pi ^{(t)}g_2(I_i|\mu _2^{(t)},\sigma _2^{(t)})} \end{aligned}$$
- M-step: compute the weighted means, variances and class probability for $j=1,2$,
  $$\begin{aligned} \mu ^{(t+1)}_j&=   \frac{\sum _{i=1}^N p^{(t)}(\Delta =j|I_i)x_i}{\sum _{i=1}^N p^{(t)}(\Delta =j|I_i)} \\ {[}\sigma ^2_j]^{(t+1)}&=   \frac{\sum _{i=1}^N p^{(t)}(\Delta =j|I_i)(x_i-\mu ^{(t+1)}_j)^2}{\sum _{i=1}^N p^{(t)}(\Delta =j|I_i)} \\ \pi ^{(t+1)}&=   \frac{\sum _{i=1}^N p^{(t)}(\Delta =2|I_i)}{N} \end{aligned}$$

**Figure 4:**
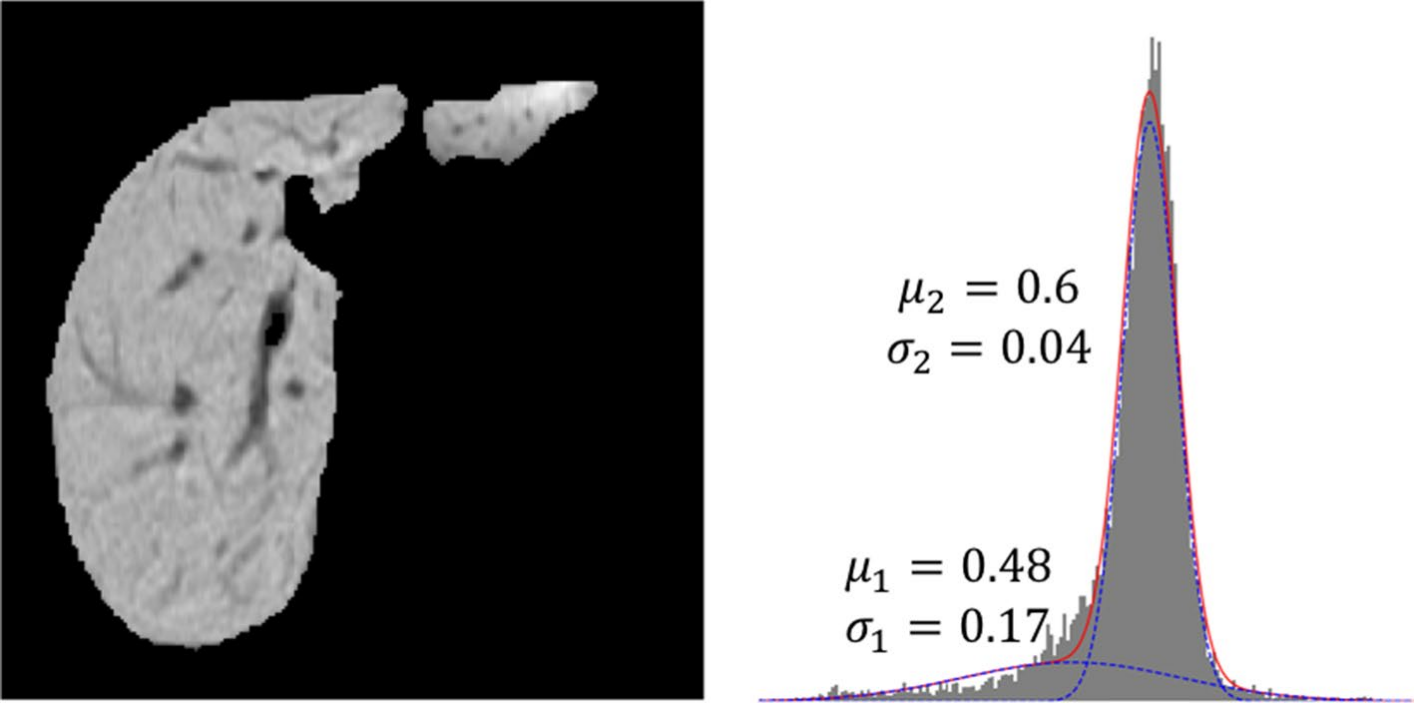
An example of GMM of one liver MR image. The mixture distribution of the image (red line) is well separated by two Gaussian distributions (blue line). The lower curve captures darker blood vessels and the upper curve captures brighter liver background tissue.

The R package mixtool^15^ was applied for this step yielding estimates of $(\mu _1,\sigma _1,\mu _2,\sigma _2,\pi )$. An example of the mixture distribution and single component distribution is offered in Fig. 4. It is important to notice that because of the standardization process in the preprocessing step, the five parameters satisfy the following constraints:

$$\begin{aligned}&\pi \mu _1 + (1-\pi ) \mu _2 =0 \\&\pi ( \sigma _1^2+\mu _1 ^2) + (1-\pi ) (\sigma _2^2+\mu _2 ^2)=1 \end{aligned}$$

In other words, the GMM does not reduce the data to five parameters but only to three. Thus, for each subject, the estimated $\mu _1$, $\sigma _1$ and $\pi$ were saved as features into the RF classifier.

#### Euler characteristic curve (ECC)

The spatial structure of liver MR images was captured by Euler characteristic (EC) curves. The EC $\psi$ is a topological quantity for many general classes of well-behaved sets^16^. For 3D Euclidean volume *S*, $\psi$ is given by,

$$\begin{aligned} \psi (S) = \#{\text { of connected components in }} S - \#{\text { of handles in }} S + \#{\text { of voids in }} S \end{aligned}$$

**Figure 5:**
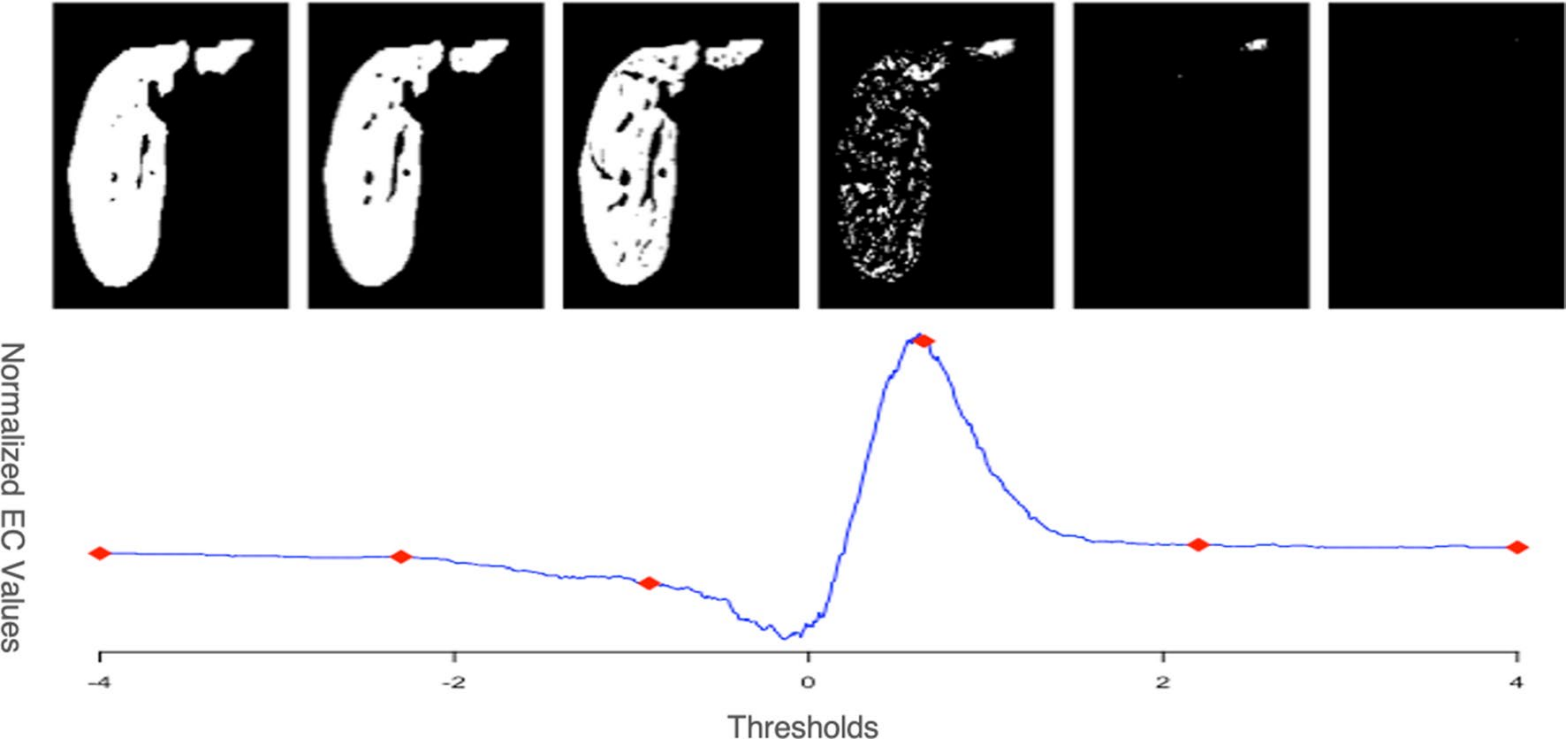
An example EC curve defined across varying thresholds and its corresponding image of excursion set.

However, for a finite simplicial complex with $d=3$, the EC can be more readily calculated using the alternative expression,

$$\begin{aligned} \psi (S) = V+F-E \end{aligned}$$

where *V*, *F*, *E* are the numbers of vertices, faces and edges, respectively. The EC curve of a grayscale image is then constructed by computing the excursion sets $A_u$ of a region *S*, defined as,

$$\begin{aligned} A_u = \{s \in S: I(s) > u\} \end{aligned}$$

where *u* is a sequence of intensity thresholds. Since the number of voxels included in each liver mask was different, we normalized the original EC value by dividing the numbers of liver voxels. Figure 5 demonstrates the construction of an EC curve for a 2D liver slice.

Richardson and Werman^17^ used the curvature of EC curve as features for objects classification. In this paper, we used methods from Crawford et al.^18^ and treated each curve as a functional input. Noticing that EC curves are piecewise-constant functions, to acquire a continuity of the inputs, we follow the work of Crawford et al.^18^ and smooth them by integrating them from right to left (positive *u* to negative *u*). Adapting ideas from functional data analysis, features from integrated curves are extracted by functional principal component analysis (FPCA)^19^.

The main idea of the FPCA is dimension reduction by means of a spectral decomposition of the covariance matrix. A smoothed EC curve *X* has moments as follows: a mean function $\mu (u) = E(X(u))$ and a covariance function $G(u, u') = Cov(X(u), X(u'))$. The covariance $G(u,u')$ can be represented as $G(u,u') = \Sigma _{k=1}^\infty \lambda _k\phi _k(u)\phi _k(u')$, allowing the curve *X*(*u*) to be expressed through the Karhunen–Loève expansion^20^,

$$\begin{aligned} X(u)&=   \mu (u) + \Sigma _{k=1}^\infty \xi _k \phi _k(u) \\ \xi _k&=   \int (X(u) - \mu (u))\phi _k(u) du \end{aligned}$$

By construction, the expansion coefficients $\xi _k$ are uncorrelated with mean 0 and variance $\lambda _k$ and are frequently referred to as functional principal component scores (FPC scores). $\phi _k(u)$ is the corresponding eigenfunction. For each subject, the first three FPC scores are treated as features extracted from the smoothed EC curves, which explains over 99% of variance of the EC curves.

#### Texture analysis (GLCM)

Texture analysis is frequently used to classify radiological images^21^. Wu et al.^22^ used texture features for classifying fibrosis stage and necroinflammatory activity in the liver. Generally, texture features from statistical approaches include histogram, gradient, gray-level co-occurrence matrix (GLCM), etc. Considering the spatial correlations between voxels, the GLCM, which describes pairwise arrangement of voxels with the same gray-level, was used in this study to extract information of local similarities.

Co-occurrences of pairs of voxels are defined using relative distance^21^. In addition, the grayscale value of each voxel is quantized to $N_g$ gray levels. Therefore, a matrix of relative frequencies consists of $P_{k,l}$, the probability of two neighboring voxels at a distance *d* and an angle $\alpha$, having the intensity scales *k*, *l* ($k,l=1,2,...,N_g$), respectively.

Haralick et al.^21^ proposed fourteen texture features extracted from the GLCM for quantitative analysis of image texture. P. Mohanaiah et al.^23^ showed that four second order features provide high discrimination accuracy in image analysis: Angular Second Moment (energy), Correlation, Entropy, and the Inverse Difference Moment (IDM). They are defined as:

$$\begin{aligned} {\text {Energy}}&=   \Sigma _{k,l}^{N_g} (P_{k,l})^2 \\ {\text {Correlation}}&=   \frac{\Sigma _{k,l}^{N_g}(k,l)P_{k,l}-\mu _k\mu _l}{\sigma _k\sigma _l} \\ {\text {Entropy}}&=   -\Sigma _{k,l}^{N_g} P_{k,l}log(P_{k,l}) \\ {\text {IDM}}&=   \Sigma _{k,l}^{N_g} \frac{P_{k,l}}{1+|k-l|^2} \end{aligned}$$

where $\mu _k = \Sigma _{k,l}^{N_g} kP_{k,l}$, $\mu _l=\Sigma _{k,l}^{N_g} lP_{k,l}$ and $\sigma _k = \Sigma _{k,l}^{N_g} P_{k,l}(k-\mu _k)^2$, $\sigma _l = \Sigma _{k,l}^{N_g} P_{k,l}(l-\mu _l)^2$. These four features were summarized as texture features for classification and extracted using the python package Radiomics^24^.

#### Deep convolutional neural network (CNN)

As an alternative to HC features, a CNN was trained to determine adequacy of HBP images. The CNN is a 50-layer residual network based on the ResNet50 architecture of He et al.^25^. Input to the CNN comprised a 224x224x10 array consisting of the same 10 liver MR image slices produced by the liver segmentation network mentioned above. A 128-neuron layer with rectified linear unit activation function was appended to the 2048-neuron feature layer from the original ResNet50 architecture to reduce the feature dimension for subsequent random forest implementation. The ResNet50 output layer was was replaced with a 2-neuron layer with softmax activation, representing the adequate and suboptimal HBP classes.

Input images were scaled to 0–1 prior to training. Optimization of model weights was performed using the gradient descent optimization algorithm with Adam stochastic optimizer using momentum terms 0.9 and 0.999. Networks for each sample size were trained using a batch size of 4 and an initial learning rate of 1e–5 with step decay. Input images were augmented using random rotations ($\pm 15$ degrees), shifts within slices ($\pm 20$ pixels) and across slices ($\pm 5$ slices), horizontal flipping, and zoom (95–110%) during training. The CNN was implemented using the Keras API^26^ and trained on a workstation with NVIDIA Titan V graphics processing unit. Following model training, input arrays were propagated through the CNN and the resulting 128 CNN features from the appended feature dimension reduction layer were extracted for subsequent random forest modeling.

### Model classifier

GMM, ECC, and GLCM features were used as inputs to a random forest (RF) classifier and implemented with the R package caret^27^. The RF consists of a large number of individual decision trees that operate as an ensemble. Each individual tree provides a class prediction and the class with the most votes is the model prediction. Considering the complexity of our selected feature spaces, RF was chosen as opposed to other classification methods because of its flexibility and accuracy^28^.

For varying combinations of features and training sample sizes, each classifier was trained with 10-fold cross validation and for each scenario, the model with the largest area under the ROC curve (AUC) was selected. Tuning hyperparameters included the number of split variables and the number of trees and the procedure was performed via the ‘train’ function from the R caret package. Model performance was evaluated using AUC and specificity at 95% sensitivity using a leave out test set (30% of all inputs). Here, sensitivity is defined as the probability of correctly classifying suboptimal HBP images. High sensitivity of 95% was enforced to ensure high detection rate of suboptimal HBP images, since incorrectly classifying a suboptimal image as adequate may prompt termination of the exam prior to reaching proper HBP, potentially impacting diagnostic value.

We also applied the RF using CNN features for a consistent comparison to HC feature performance. Although the final layer of a CNN predicts labels automatically, posthoc modeling of CNN features using other classifiers has shown improvements in predictive performance^29,30^. For computational tractability of the RF, we appended an additional 128-neuron layer to the 2048 feature layer of the original ResNet50 architecture to reduce the CNN feature dimension to a manageable number of features. The 128 neurons were saved as CNN features for the RF classifier, maintaining the prediction ability ($\hbox {AUC}=0.93$). When implementing the CNN directly as the classifier, the full sets of features and reduced sets of features yielded the same values of AUCs. Meanwhile, the computation time with the RF classifier was tremendously decreased with the lower-dimensional feature space.

## Results

### Model performance with complete training data

**Figure 6:**
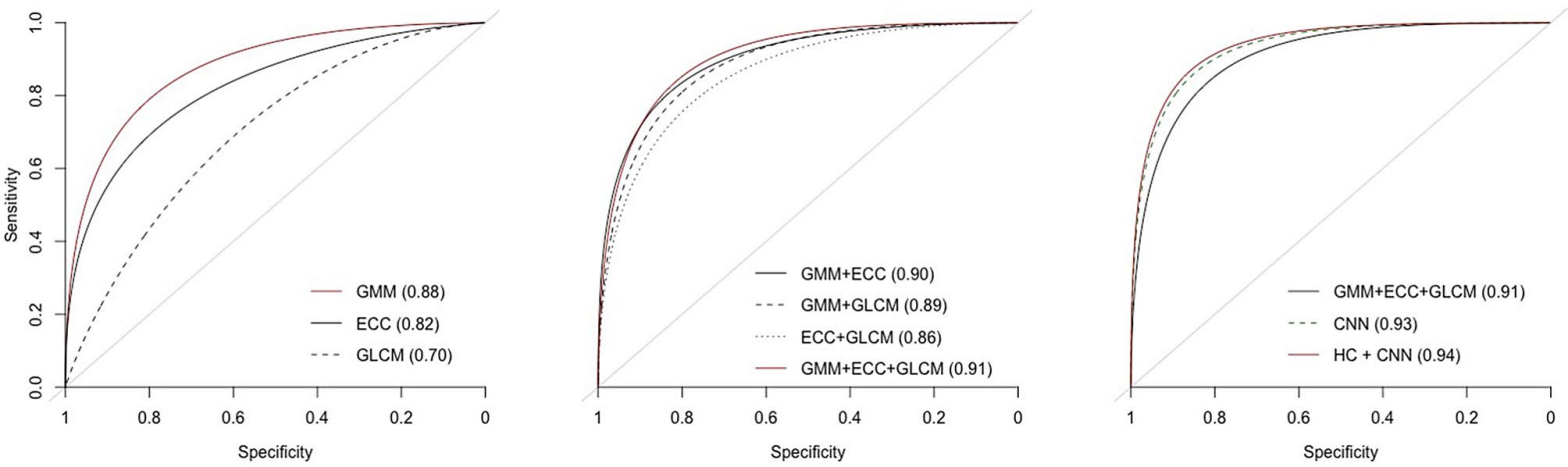
ROC curve and AUC value of testing data incorporating different combination of feature inputs trained with full-size training data.

Figure 6 shows the AUCs of testing set from RFs with different combinations of features, trained with the complete training set (826 images). The detailed results are shown in Table 1 and the 95% confidence intervals are computed based on 2000 bootstraps replicates on the single testing set. GMM yields the best prediction performance ($\hbox {AUC} = 0.88$) among the three univariate HC models while GLCM has the worst prediction performance ($\hbox {AUC} = 0.70$). Models with two HC features perform similarly if GMM is included (AUC $\approx 0.89$), while the combination of ECC and GLCM has the worst performance. AUC increases to 0.91 using all three HC features, which is slightly smaller in magnitude to the model with CNN features only. Combining HC and CNN features outperforms all other models ($\hbox {AUC} = 0.94$).

**Table 1:** AUC and specificity (with sensitivity = 0.95) of testing data for models with different combinations of features using RF with the complete training data.

Features	AUC	95% Confidence interval	Specificity	95% Confidence interval
GMM	0.88	0.840–0.913	0.49	0.373–0.609
ECC	0.82	0.775–0.865	0.30	0.203–0.420
GLCM	0.70	0.636–0.754	0.22	0.138–0.310
GMM + ECC	0.90	0.866–0.936	0.55	0.440–0.676
GMM + GLCM	0.89	0.851–0.921	0.55	0.425–0.640
ECC + GLCM	0.86	0.821–0.902	0.45	0.340–0.578
GMM + ECC + GLCM	0.91	0.873–0.941	0.62	0.514–0.736
CNN	0.93	0.904–0.957	0.69	0.600–0.787
ALL	0.94	0.913–0.961	0.72	0.612–0.803

Specificities are computed at 95% sensitivity. For univariate HC models, GMM has the largest specificity (0.49) among all three features. For two-variable models with HC features, specificities of all three models improves. The model incorporating all HC features further improves the specificity to 0.62. Compared with the specificity of the CNN-feature model (0.69), the value is increased to 0.72 when using CNN and HC features in the RF model.

### Model performance with partial training data

With concerns regarding sample size constraints in medical imaging, we performed comparisons using different sizes of training data. The best three models described in the prior section were implemented, including the ones using all HC features, CNN features and their combination.

**Figure 7:**
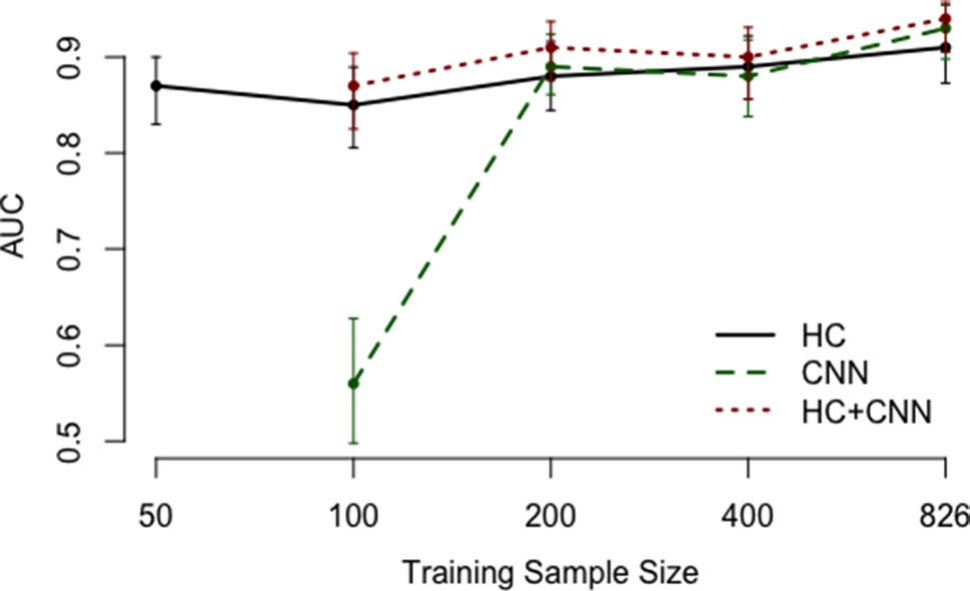
Testing AUC comparison using different sizes of training data, with 95% confidence intervals.

As shown in Fig. 7, the model using HC features is quite robust to training sample size *n* and consistently produces AUCs from 0.85 to 0.91 with increasing training sample size. Conversely, the CNN was unable to converge with $n=50$. At $n=100$, CNN AUC is around 0.58, suggesting the model has little or no ability to separate classes. With increasing *n*, the CNN achieves better performance ($\hbox {AUC} = 0.88$ for $n=400$). Finally, combining CNN features with HC features improves model performance over HC and CNN only features across different training sample sizes.

### Variable importance analysis

**Figure 8:**
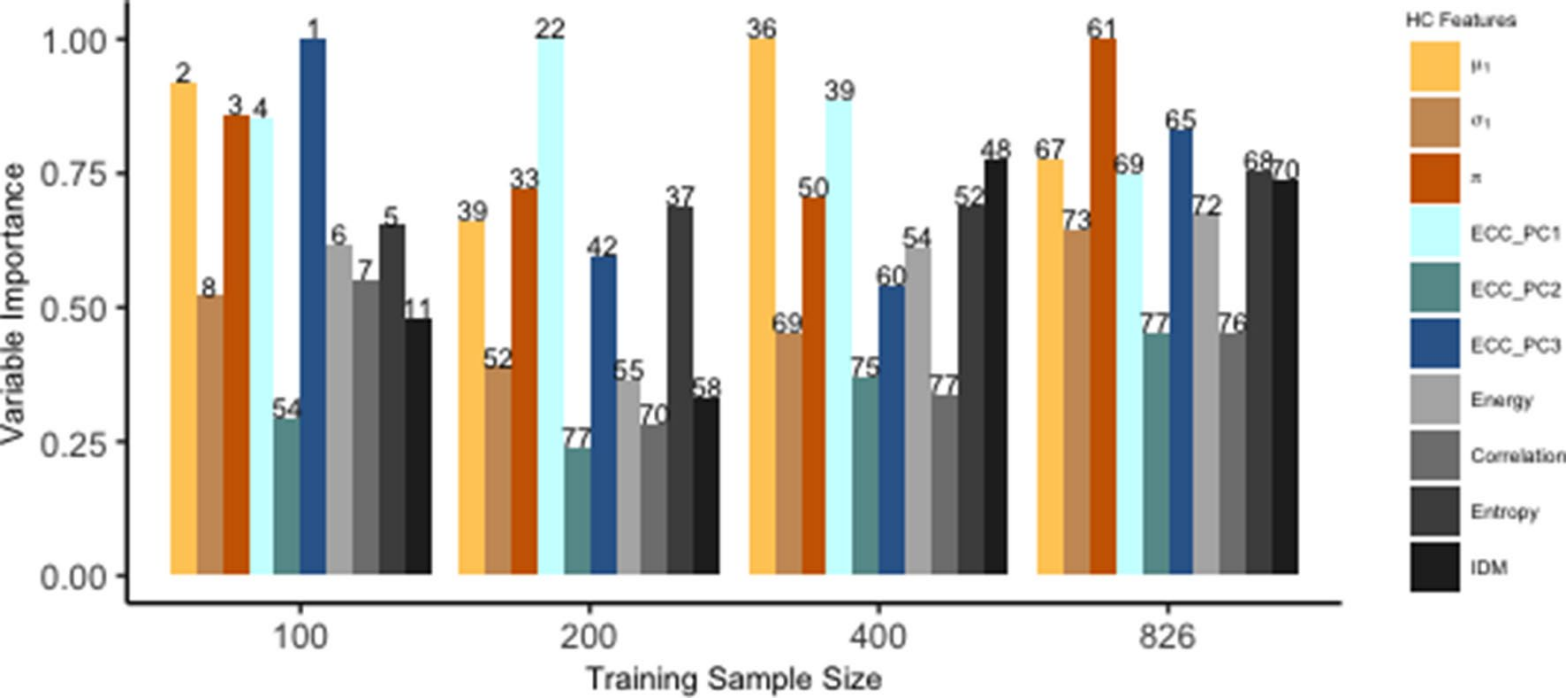
Variable importance summary of HC features in RF classifiers with both HC and CNN features included. Numbers above color bars represent ranks of importance among all features.

Figure 8 summarizes the values of Mean Decrease Gini (MDG) of each HC feature from the RF classifiers including both HC and CNN features, trained with different sample sizes. The MDG measures the averaged total decrease in node impurities from splitting on the variable and describes the impact of each predictor variable individually as well as in multivariate interactions with other predictor variables^31^. The MDG is computed from the R package caret and larger MDG implies higher variable importance. With decreasing training sample sizes, HC features tend to have higher ranks among all features and present more impact in the RF classifier. In addition, the relative importance for HC features has a similar pattern across different training sample sizes with the model including only HC features (not shown). Some features, such as $\mu$ (weighted mean), $\pi$ (class weight) from the GMM and the first principal component from the ECC, consistently have relatively more importance in model prediction.

## Discussion

In this paper, we aimed to develop and compare machine learning approaches for automatically classifying liver MR images as either having suboptimal or adequate HBP. With comparable results based on AUC values, we conclude that a classifier, with either HC features or CNN features, is able to distinguish between adequate and suboptimal HBP liver MR images. Additionally, we find that when trained with all available data, performance of CNN features and HC features are comparable while the combination of CNN and HC features yields the best model performance (AUC = 0.94). With this finding, when acquisition of large datasets for training purposes is not an issue, combining HC and CNN features can improve model performance, demonstrating that HC and CNN features can extract different information from the original liver MR images.

In addition to AUCs, we compared the specificity of each model at 95% sensitivity trained with full datasets. These results are consistent with AUCs, i.e. the combination of HC features and CNN features yields the highest specificity. A high sensitivity value of 95% was chosen because real time assessment of HBP adequacy is important to avoid termination of the exam before adequate liver uptake is achieved, which may improve diagnostic accuracy and reduce the need for patient recall and rescanning. Since misclassification of suboptimal images has the greatest clinical impact, we evaluated our methods using a high sensitivity of 95% to ensure suboptimal images are accurately detected. This effectively controlled the level of type-II error for classifying suboptimal HBP as adequate.

**Figure 9:**
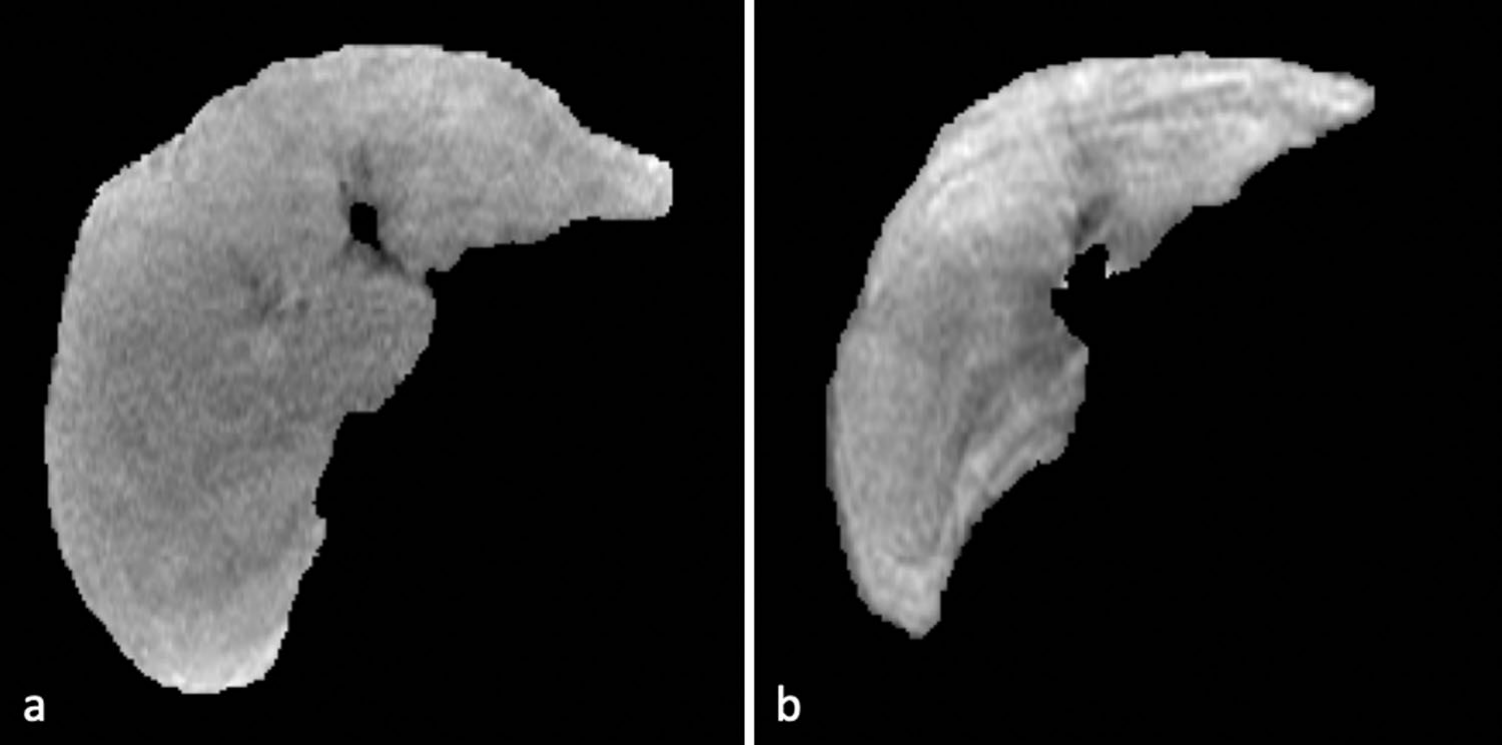
Examples of liver images misclassified as adequate using (**a**) ECC features and (**b**) GMM features.

**Figure 10:**
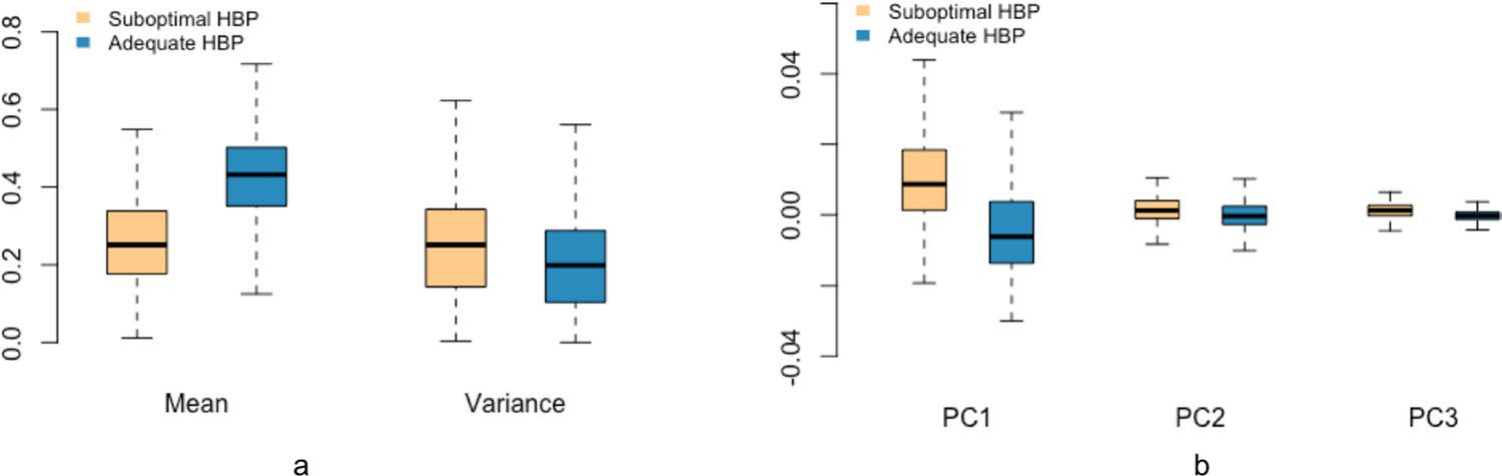
Overall distribution of features extracted from suboptimal and adequate HBP liver MR images with (**a**) GMM, (**b**) ECC.

Another advantage of HC features is interpretability. GMM features address the problem of voxel intensity separation and ECC features are used for extracting topological patterns. Two examples of misclassification with these two types of features are shown in Fig. 9. In (a), the image is correctly classified as suboptimal by GMM features alone, showing almost no contrast between vessels and background tissue due to impaired contrast uptake. The same image is misclassified as adequate when using ECC features alone, recognizing a relatively consistent spatial structure of the liver background. In (b), ECC features capture topological ambiguity and the image is correctly classified as suboptimal. The same image is mislabeled as adequate HBP by GMM features, which captures the intensity discrepancy regardless of vessels in the liver background. The distribution of extracted features from these two methods also presents clear separation between adequate and suboptimal HBP images (Fig. 10). Therefore, interpretability of HC features reveal why liver MR images are classified as suboptimal or adequate.

Although the RF classifier, consisting of a large number of deep trees, is typically treated as a black box, we used variable importance to determine which HC features contribute most to the prediction. We find that HC features rank the highest among all features when training samples are limited. Features’ importance declines in rank for larger training samples, but their patterns of relative importance remain consistent. Despite the complex interactions between HC and CNN features modeled by the RF classifier, variable importance allows us to maintain a degree of interpretability of the HC features.

In addition to overall model performance, we performed a secondary analysis to assess the effect of training sample size on the performance of HC and CNN features. It is known that CNNs require a large amount of training data for imaging classification. Here we compare the model performance with quantitative results and demonstrate that the CNN will not achieve satisfactory performance unless trained with a large sample of data, suggesting that HC features may still be needed in practice. In clinical studies, the recruitment of a large number of patients or collection of large number of images is often impeded by patient privacy, limited number of disease cases, restricted resources, funding constraints or number of participating institutions. Therefore, large datasets for CNN model training may not be readily available.

In contrast, HC features yield consistently high AUCs with limited sample sizes in our study, meaning that with robustness to training sample size, HC features can be helpful with the early stages of a study and give guidance for subsequent analyses. Furthermore, HC features are defined in advance, and therefore typically do not require large datasets for training. Hence, when the collection of large datasets is not readily practicable, a classifier implemented with HC features can still be used as a preliminary reference.

In comparison with the commonly used texture features from the GLCM, we introduced the ECC as an improvement in this paper. Texture analysis has been applied to medical images since 1973^21^ and describes the quantitative relations of intensity contrast between voxels. The ECC, however, is a more recently developed measurement of topological features^16^ and extracts information of shape and connectivity in the images. From our analysis, ECC consistently outperformed texture features and can be readily visualized for interpretation.

Still, there are limitations in our current study. Some liver MR images labeled as suboptimal HBP by radiologists could not be correctly classified by any HC features. Other HC features such as morphological features can be explored to explain image information that was not addressed in the current work. Further work should focus on investigating these features and understanding their relevance for image classification. Another limitation is the reliance on only ten slices per liver MR series due to the long computation time applying ECC in 3D. A new faster way of computing 3D ECC is under development by the authors and will dramatically increase the efficiency of the existing algorithm. Furthermore, in this paper we only addressed the question of identifying the adequacy of liver MR images. The methods and experiments must be further implemented and tested on other sources of data to further evaluate generalizability of the methods proposed.

## Conclusion

We have demonstrated the feasibility and interpretability of HC features in evaluating HBP adequacy of liver MR images, compared with the popular CNN models. With a relatively smaller size of training samples, our HC features outperform CNN features for the task of classifying HBP images as adequate or suboptimal. CNN features achieve greater classification performance as the size of training data increases. Combination of HC features and CNN features is the most favorable model under all circumstances.

## Data Availability

Liver MR images are not available for public access regarding patient privacy concerns but are available on reasonable request from the corresponding author. The code for analysis will be available upon request.
